# Design, Preparation and Performance Study of On-Chip Flow-Through Amperometric Sensors with an Integrated Ag/AgCl Reference Electrode

**DOI:** 10.3390/mi9030114

**Published:** 2018-03-07

**Authors:** He Zhang, Rongyan Chuai, Xin Li, Bing Zhang

**Affiliations:** School of Information Engineering and Science, Shenyang University of Technology, Shenyang 110870, China; me_sut@163.com (R.C.); lixin97@163.com (X.L.); zhangbingsut@163.com (B.Z.)

**Keywords:** microfluidic chip, amperometric detection, integrated Ag/AgCl reference electrode, detection cell structure optimization, mass transfer

## Abstract

To improve the reference potential stability of on-chip amperometric sensors, we propose a novel integrated Ag/AgCl reference electrode structure. This structure can refresh the saturated potassium chloride filling solution surrounding the Ag/AgCl electrode. We then designed a flow-through amperometric sensor and a multilayer microfluidic chip based on the integrated reference electrode. In order to improve the detection signal strength of the flow-through sensor, a numerical simulation model was established. The simulation results showed that a combination of (1) using a step-type detection cell structure that maintains micro-channel width while reducing micro-channel height, and (2) controlling the sample flow rate to limit the mass transfer of the sensor surface effectively, improves the detection signal strength. The step-type detection cell structure had dimensions of 200 μm × 200 μm × 100 μm (length × width × height), and the electroosmotic flow driving voltage was 120 V/cm. Finally, successful trace detection of Mg^2+^ and Pb^2+^ in the water was achieved using the amperometric sensor and microfluidic chip: detection limits were 5 μmol/L and 84 μmol/L. The preparation of an on-chip flow-through amperometric sensor with an integrated Ag/AgCl reference electrode will facilitate improved portability of microfluidic detection technology.

## 1. Introduction

Achieving rapid and accurate detection of trace samples is the ultimate goal of most microfluidic chips [1]. Due to the limitation of microfluidic chip volumes, it is necessary not only to consider common technical indicators (sensitivity, resolution and reproducibility) when selecting the detection method; rather, the volume of the sensor, the difficulty of the integration process and other factors should also be taken into account. Mass spectrometry detection [2], spectral detection [3] and electrochemical detection [4,5,6] are the three most widely used detection methods in microfluidic chips. The amperometric detection method [7,8,9] is a subtype of electrochemical detection methods, and can determine the sample components by using the redox reaction on electrodes. The advantages of this method not only include sensitivity and versatility, but integration and miniaturization capability as well, making it suitable for use in microfluidic chips [10,11].

Amperometric detection sensors in microfluidic chips usually consist of one working electrode, one auxiliary electrode and one reference electrode. The sensor is often placed in a special detection cell or the end of a micro-channel [12]. Due to their functional differences, the structure, material type and processing technology of these electrodes can also vary significantly. The Ag/AgCl reference electrode, which consists of one Ag electrode covered by a AgCl layer which is then immersing in KCl solution (Ag|AgCl|Cl^−^), can provide a stable reference potential for amperometric sensors. However, compared to the working electrode and the auxiliary electrode that can be prepared with precious metals such as Pt and Au, the integration of Ag/AgCl reference electrodes is more difficult [13,14,15]. Although the solid-state Ag/AgCl reference electrode [16] has been successfully integrated on-chip, the concentration of Cl^−^ ions in the solid film is limited. Thus, the stability of the reference potential cannot be guaranteed after repeated use. Moreover, the AgCl layer can easily fall off when exposed in a dry environment.

In this paper, a novel integrated Ag/AgCl reference electrode structure was proposed. During on-chip amperometric detection, the potential stability of the novel reference electrode can be guaranteed by updating the saturated potassium chloride filling liquid and replacing the Ag/AgCl electrode. Based on the integrated reference electrode, a flow-through amperometric sensor and multilayer microfluidic chip were designed. Then, a simulation model of the mass transfer diffusion on the sensor surface was established to optimize the detection cell structure and improve detection signal strength. According to the optimization results, a microfluidic chip with an amperometric sensor was fabricated and characterized.

## 2. Sensor Design and Optimization

**Figure 1:**
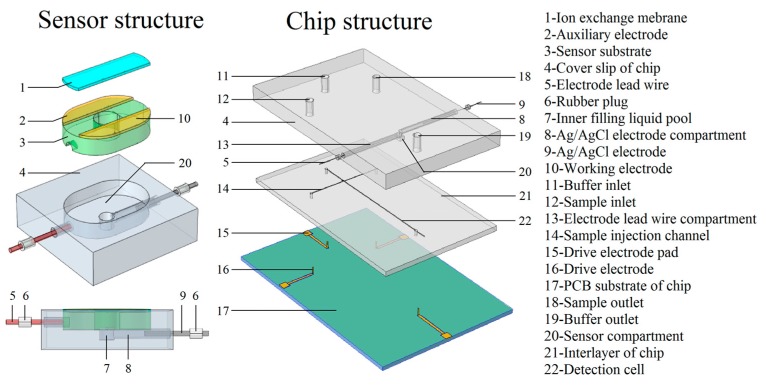
Amperometric sensor and microfluidic chip structure.

As shown in Figure 1, in order to integrate the Ag/AgCl reference electrode, the cover slip thickness of the microfluidic chip was increased. On the thickened cover slip, an inner liquid pool was designed to be filled with saturated potassium chloride solution. Then the Ag/AgCl electrode was inserted into the compartment, and sealed with a rubber plug, creating an embedded reference electrode. This structure allows Cl^−^ ions to be replenished when the Cl^−^ ion activity is insufficient or when the AgCl coating fails. Based on the embedded Ag/AgCl reference electrode, the flow-through amperometric sensor and multilayer microfluidic chip were designed. The chip consisted of three layers: the PCB (Printed Circuit Board) substrate with an electroosmotic flow drive electrode; the interlayer with the micro-channel and detection cell; and the cover slip with the amperometric sensor, inner filling liquid pool, sample inlet and so on. During chip testing, the flow direction was parallel to the sensor surface: the samples can update continuously to ensure the redox reaction on the sensor surface.

Although, the on-chip amperometric detection involves a series of complex electrochemical reaction steps, once the electrode material was determined and the potential stability of the reference electrode was guaranteed, the detection signal strength was mainly determined by the mass transfer process at the “electrode-sample” interface [17]. In order to investigate the mass transfer diffusion on the sensor surface, a numerical simulation model was established in COMSOL Multiphysics. The model was based on the hydrodynamic equation and mass transfer diffusion equation. The initial parameters of the model are shown in Table 1.

**Figure 2:**
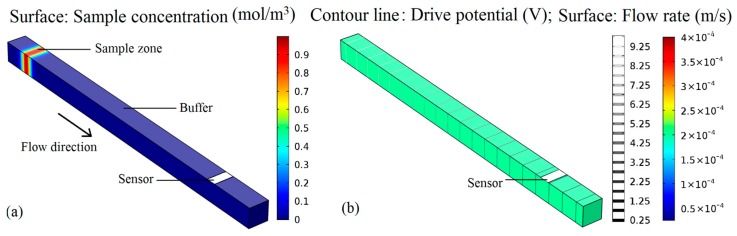
Microchannel mass transfer model driven by electroosmotic flow: (**a**) Surface concentration, (**b**) Fluid velocity and driving potential distribution.

The sample zone concentration distribution in the micro-channel from the initial case (*t* = 0) is shown in Figure 2a. When the drive potential was applied on the micro-channel, the sample zone would move toward the sensor because of the electro-osmosis flow. The drive potential distribution and fluid velocity are shown in Figure 2b. It is evident that the drive potential is distributed evenly on the rectangular channel, and the flow velocity is constant at around 2 × 10^−4^ m/s. The calculated Reynolds number (*Re*) is 0.025, which is far less than 1. Thus, the fluid flow in the micro-channel is typical laminar flow.

**Figure 3:**
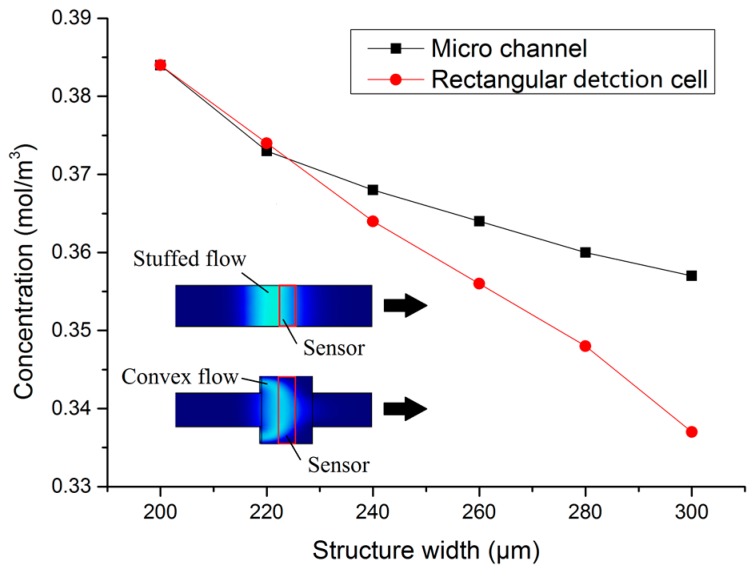
Working electrode width variation versus surface mass transfer.

Figure 3 shows how the sample concentration changes in response to changing the area of the working electrode surface (by increasing the width of either the micro-channel or the detection cell). When the initial concentration was 1 mol/m^3^ and the working electrode surface area was *W* × *L* = 200 × 100 μm^2^, the maximum concentration of the sample zone was 0.384 mol/m^3^; when the working electrode surface area increased to *W* × *L* = 300 × 100 μm^2^ (micro-channel widening), the maximum concentration of the sample zone was 0.357 mol/m^3^, a reduction of 7.0%. When the working electrode surface area was *W* × *L* = 300 × 100 μm^2^ (detection cell width increase), the maximum concentration of sample zone was 0.337 mol/m^3^, a reduction of 12.2%. The sample concentration on the surface decreased as the working electrode widened. While widening either the micro-channel or the detection cell width resulted in the same increase in working electrode area, widening the detection cell led to a greater decrease in the sample zone concentration. This is because increasing the electrode surface area through increasing the width of the micro-channel also increases the contact area between the sample zone and buffer solution. Increasing the contact area accelerates the mass transfer diffusion rate, which results in the reduction of the sample zone concentration. Conversely, the pressure gradient in the micro-channel changes in response to sudden widening at the detection cell. As a result, the sample zone flow changes from stuffed flow to convex flow. The interface area of contact between the sample zone and the buffer rapidly increases due to convex flow, which promotes mass transfer diffusion, hence the concentration drops faster. Meanwhile, the trailing phenomenon (also caused by the convex stream) could result in the overlapping of detection signals from different samples. Therefore, when designing microfluidic chips based on flow-through amperometric sensors, the working electrode width should be consistent to prevent the lateral expansion of the sample zone.

**Figure 4:**
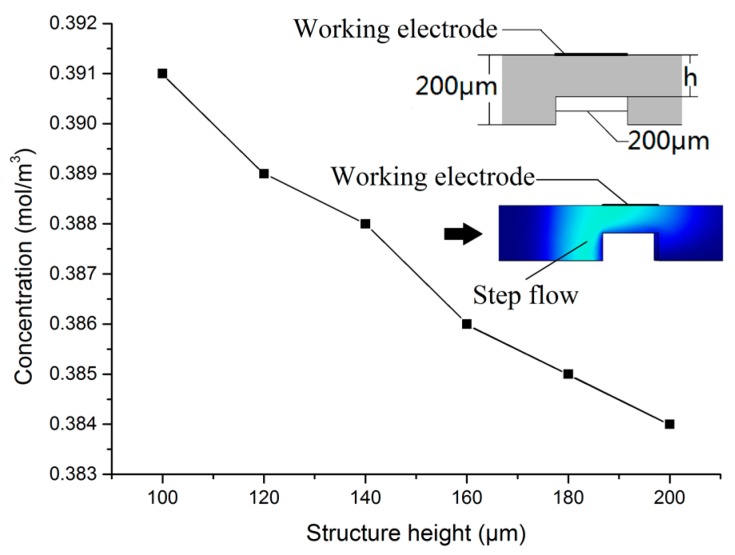
Micro-channel height versus mass transfer of working electrode surface.

The micro-channel height versus working electrode surface mass transfer is shown in Figure 4 (in this simulation micro-channel width was held constant (200 μm), and a step structure was added under the sensor). As shown in the figure, when the micro-channel height was 200 m, the maximum concentration of the sample zone on the working electrode surface was 0.384 mol/m^3^; whereas when the micro-channel height was 100 μm, the maximum concentration was 0.391 mol/m^3^. The detection micro-channel height is, thus, inversely proportional to the sample zone concentration. When the micro-channel height is reduced by 50%, the max concentration increased by 1.6%. This is because the reduction in micro-channel height reduces the longitudinal contact area between the sample zone and buffer solution, which can limit the mass transfer rate between them.

**Figure 5:**
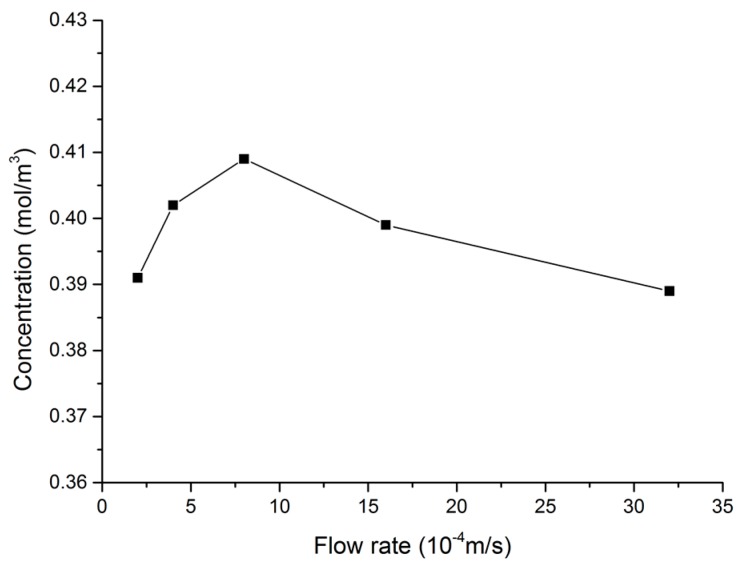
Velocity variation versus mass transfer of the working electrode surface.

Finally, the effect of flow velocity on electrode surface diffusion mass transfer was investigated, for the case of a step-type test detection cell structure with a structure height of 100 μm. As shown in the Figure 5, when the flow velocity was 2 × 10^−4^ m/s, the maximum concentration of the sample zone was 0.39 mol/m^3^. When the sample flow velocity increased to 8 × 10^−4^ m/s, the maximum concentration of the sample zone was 0.409 mol/m^3^, an increase of 4.6%. At this time, the driving voltage applied to the micro-channel was about 114 V/cm. When the flow velocity increased to 32 × 10^−4^ m/s, the maximum concentration of the sample zone was 0.38 mol/m^3^, a reduction of 0.5%. As the flow velocity increases, the time it takes for the sample to reach the electrode surface decreases, so the maximum concentration increases. However, when the flow velocity is further increased, step flow occurs near the surface of the working electrode because of the step structure detection chamber [18]. The step flow would increase with the increase in flow velocity. The perturbation that is generated by the step flow would promote mass transfer between the sample and the buffer diffusion. Thus, an excessively large flow velocity is harmful when trying to limit mass diffusion at the electrode surface. The chip driving voltage should be around 114 V/cm.

## 3. Sensor and Microfluidic Chip Preparation

**Figure 6:**
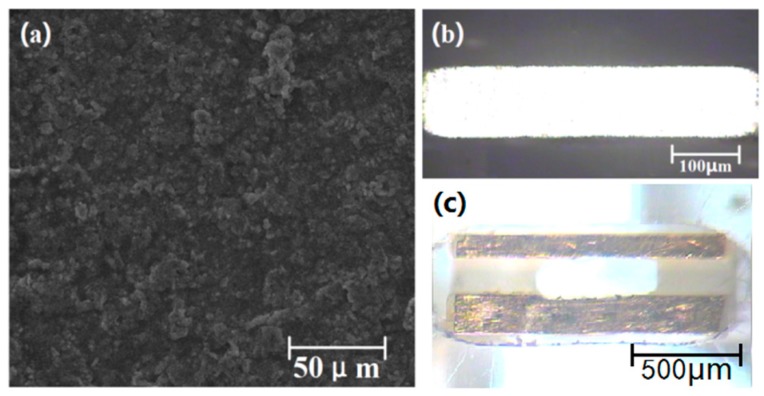
(**a**) Ion exchange membrane, (**b**) Printed Circuit Board (PCB) electrode and (**c**) amperometric sensor.

The flow through the amperometric sensor and the microfluidic chip preparation process included: embedded reference electrode preparation, working electrode and auxiliary electrode preparation, microfluidic chip preparation and assembly. The embedded reference electrode preparation was as follows: First, the ion exchange membrane was prepared. The Cl^−^ exchange membrane of the reference electrode was fabricated from polyvinyl chloride (PVC). Saturated potassium chloride, PVC powder and o-nitrophenyl ether plasticizer were dissolved in a freshly distilled solution of tetrahydrofuran (mass ratio of 5:32:63). After standing for 24 h at room temperature, a PVC chloride ion exchange membrane formed (Figure 6a). Second, the Ag/AgCl electrode was prepared. This was constructed as a reaction system using silver wire as the working electrode, platinum wire as the auxiliary electrode, and the standard Ag/AgCl electrode as a reference electrode. These wires were then connected to the corresponding port of the electrochemical workstation for 180 seconds under PVI mode. Thus, the Ag/AgCl electrode was obtained. Third, the testing reference electrode was prepared. The PVC chloride ion exchange membrane was cut into discs (*r* = 3mm) and glued to one end of a PVC tube with tetrahydrofuran solution. The Ag/AgCl electrode was inserted into the other end of the PVC tube and filled with saturated potassium chloride solution and sealed. 

The working and auxiliary electrodes of the amperometric sensor were fabricated using the PCB process. The preparation process is now briefly described. *PCB electrode preparation:* the electrode structure diagram was designed using Protel DXP, and externally machined. The resulting electrode was gold-plated. The gold-plating solution was composed of Na_3_Au(SO_3_)_2_ (7 × 10^−3^ mmo1/L), Na_2_SO_3_ (1.25 × 10^−3^ mo1/L), HCHO (0.6 mo1/L). The PCB electrode was immersed in the solution, and the reaction conditions were set to complete the gold-plating process (pH: 10–12, temperature: 2–5 °C). The prepared electrode is shown in Figure 6b. *Sensor assembly:* an Ultra-precision engraving machine was used to produce the micro-structure between the working electrode and the auxiliary electrode. The PVC mixed solution was poured into the microgrooves to form the ion exchange membrane. The completed amperometric sensor is shown in Figure 6c.

**Figure 7:**
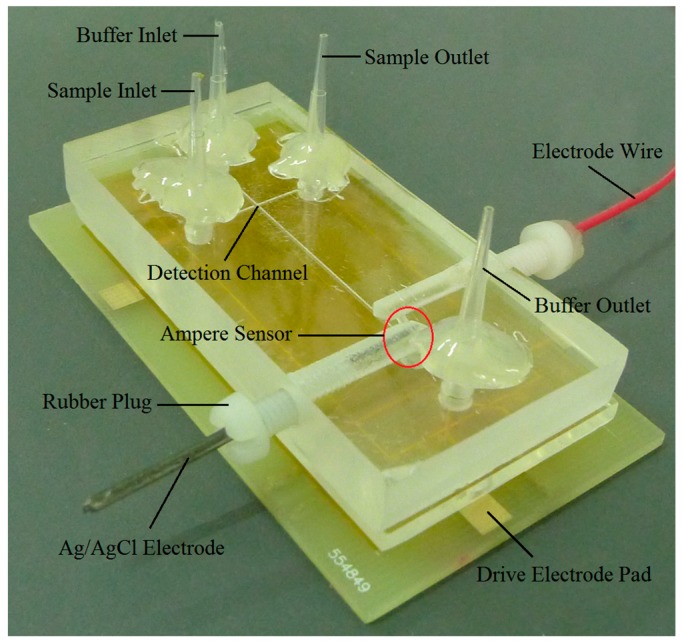
Microfluidic chip layout.

According to the chip structure design displayed in Figure 1, the preparation process of the amperometric detection microfluidic chip was as follows: the chip substrate was fabricated using PCB. The microstructure was formed on polymethyl methacrylate (PMMA) with an ultra-precision engraving machine; the electrodes leads were welded and fitted to the cover sheet. The electrodes were bonded to the PMMA via organic solvent immersion [19]. The gold drive electrode was welded to the PCB, and the PMMA middle layer was bonded to the PCB using modified acrylic adhesive. KCl solution was injected into the inner filling pool and the Ag/AgCl electrode was inserted and then sealed with hot melt glue and a rubber plug. The prepared chip is shown in Figure 7.

## 4. Results and Discussion

**Figure 8:**
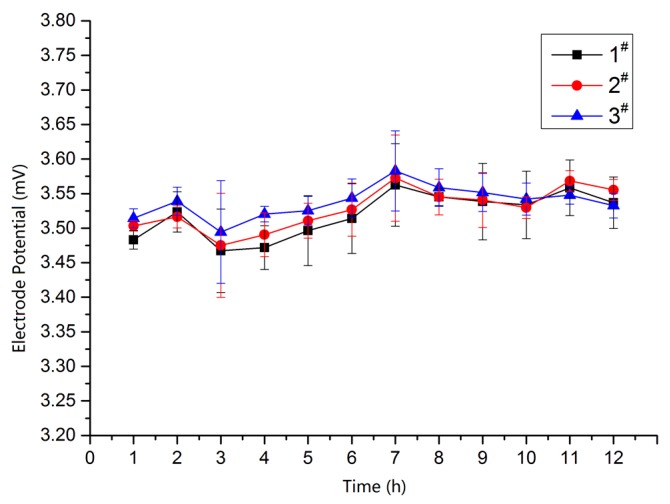
Reference electrode potential versus time.

**Figure 9:**
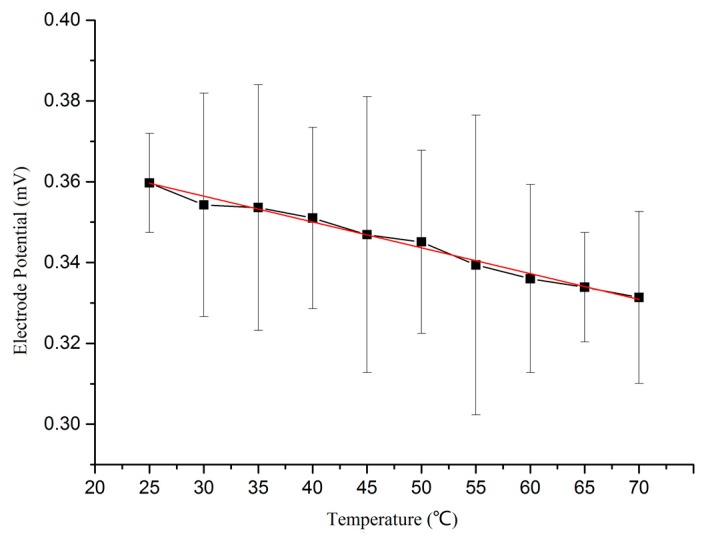
Reference electrode potential versus temperature.

The testing of amperometric sensor was divided into two parts. First, the stability and temperature response of the self-made Ag/AgCl electrode were characterized to ensure reference potential precision. Next, the overall performance of the sensor was characterized using the assembled chip. The specific steps testing in testing the stability and temperature response are as follows:

1. Detection of reference electrode stability. Three self-made Ag/AgCl reference electrodes were labelled as 1^#^, 2^#^ and 3^#^, then immersed into KCl solution (0.0l mol/L) at room temperature (25 °C). The stability of the electrodes was measured using an electrochemical workstation. The test results are displayed in Figure 8, The potential drifts of the three electrodes are all within 0.10 mV, indicating good stability for all three self-made Ag/AgCl reference electrodes.
2. Detection of the temperature response. A testing reference electrode was immersed into KCl solution (0.0l mol/L), and the temperature was adjusted using an oven. The potential of the self-made Ag/AgCl reference electrode versus a standard saturated calomel electrode (SCE) was measured using an electrochemical workstation. The testing curve is shown in Figure 9. It can be seen from the curve that the electrode potential is inversely proportional to the temperature. The electrode potential was reduced from 0.359 mV to 0.331 mV, when the temperature rose from 25 °C to 70 °C. The expression E_Ag/AgCl_ = 0.376 − 6.389 × 10^−4^·*T* that was obtained by using the software Origin9.0 (OriginLab, Hampton, MA, USA). The electrode reaction of Ag/AgCl can be written as:
  (1) $$\mathrm{AgCl}+e^{-}↔\mathrm{Ag}+{\mathrm{Cl}}^{-}$$

Therefore, according to the Nernst equation, the electrode potential of the Ag/AgCl reference electrode versus Cl^−^ can be written as

(2) $$E_{\mathrm{Ag}/\mathrm{AgCl}}=E^{0}-\frac{RT}{\mathrm{nF}}\ln\alpha_{{\mathrm{Cl}}^{-}}$$

where, *E*^0^ is the standard electrode potential; R = 8.314 J/K·mol is the gas constant; *T* is the thermodynamic temperature; *F* = 96485 J/mol·V is the Faraday constant; *n* is the number of electrons transferred in the reaction; and *α*_Cl_^−^ is the activity of Cl^−^ in the reaction, which can be replaced by the concentration of Cl^−^ for convenient calculation.

The theoretical value of the temperature coefficient is 5.95 × 10^−4^, which was obtained using Equation (2). However, the measured temperature coefficient was 6.389 × 10^−4^, relatively close to, but not consistent with, the theoretical value. There are two reasons for this deviation, the first is that the potential of the SCE also changes with temperature during testing; the second is that the activity of Cl^−^ was replaced by the concentration when making calculations.

**Figure 10:**
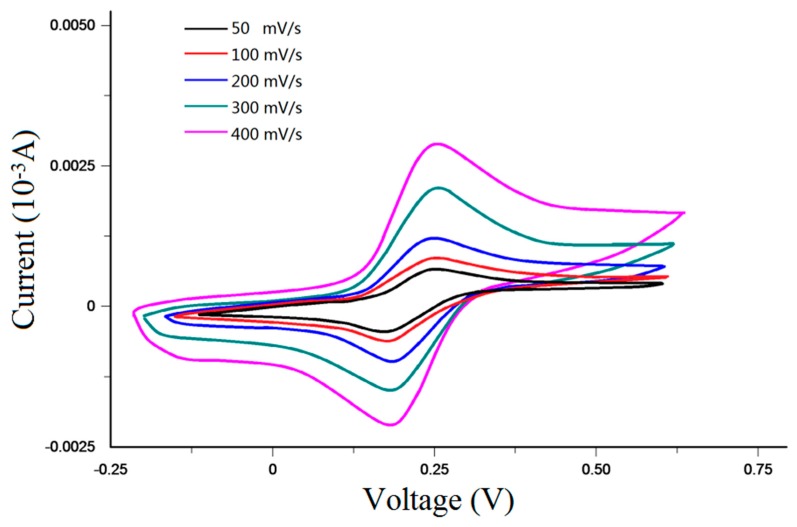
Cyclic voltammetry curve of amperometric detection chip.

**Figure 11:**
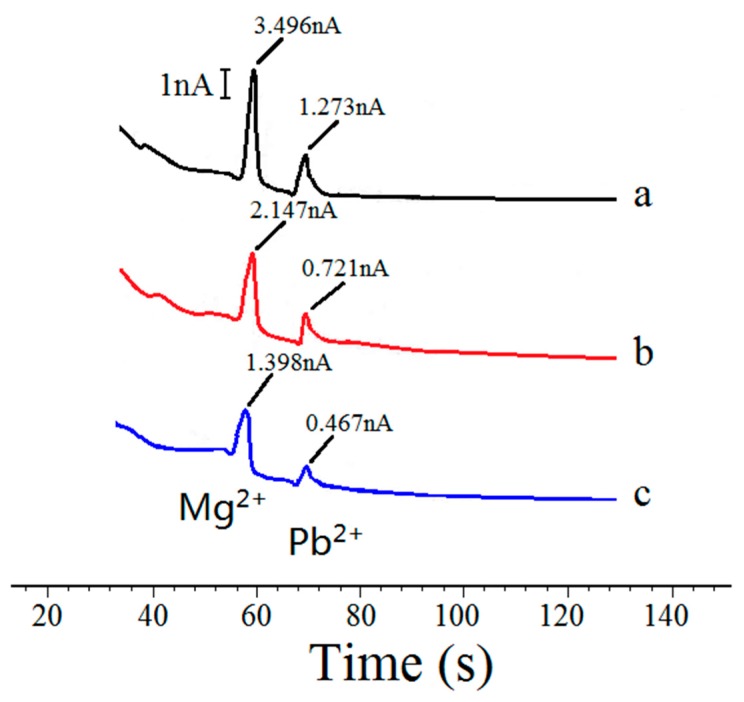
Separation detection curve of Mg^2+^ and Pb^2+^. (a) Mg^2+^ 2 mmol/L, Pb^2+^ 2 mmol/L; (b) Mg^2+^ 1 mmol/L, Pb^2+^ 1 mmol/L; (c) Mg^2+^ 0.5 mmol/L, Pb^2+^ 0.5 mmol/L.

The overall performance of the sensor was then characterized using the assembled chip. The specific testing steps are as follows:

1. Cyclic voltammetry testing. The testing solution consisted of KCl (0.1 mol/L), K_4_Fe(CN)_6_ (1 × 10^−3^ mol/L) and K_3_Fe(CN)_6_ (1 × 10^−3^ mol/L). PB buffer solution was used to adjust the testing solution pH to 5. Next, the testing solution was injected into the detection channel of the microfluidic chip, and care was taken to ensure that the integrated sensor was connected to the appropriate port of the electrochemical workstation. The start scan potential of the electrochemical workstation was −200 mV, the ending scan potential of the electrochemical workstation was 600 mV, and the scan interval was 2 mV. The cyclic voltammetry curves of the sensor for scan rates of 50, 100, 200, 300, 400 and 500 mV/s are shown in Figure 10. As shown in the figure, the oxidation/reduction peak of the sensor is approximately symmetrical, which indicates that the amperometric sensor has good reversibility.
2. The detection of heavy metal ions in water. Tricine-MES mixed solution was used as a buffer solution (20 mol/L, pH = 5), Mg^2+^ and Pb^2+^ mixed solutions of different concentrations were used as samples. The drive electrode pads of the chip were connected to a voltage power supply, and the injection voltage was set as +1000 V, while the separation voltage was set as +1200 V. The integrated sensor was connected to the appropriate port of the electrochemical workstation and the detection potential of the electrochemical workstation was set as −0.4 V. Due to the electroosmotic flow that was generated by the external electric field, the sample zone, sandwiched between the buffer solutions, moves toward the amperometric sensor. The speeds of Pb^2+^ and Mg^2+^ are different under the same driving electric field intensity. Therefore, after passing though the same length of detection channel, Pb^2+^ and Mg^2+^ ions reach the sensor surface at different times. When Pb^2+^ and Mg^2+^ ions arrive at the sensor surface, a redox reaction occurs on the golden working electrode, causing the peak current to be generated. The peak current intensity is positively correlated with Pb^2+^ and Mg^2+^ concentration, so the ion concentrations can be determined from the peak current intensity. The detection curves of Mg^2+^ and Pb^2+^ at different concentrations are shown in Figure 11. It can be seen that the sensor and the chip can achieve the separation and detection of Mg^2+^ and Pb^2+^ ions, and the detection signal intensity was proportional to the sample concentration. When the concentration of Mg^2+^ and Pb^2+^ were both 2 mmol/L, the peak currents were 3.496 nA and 1.273 nA, respectively. When the concentration of Mg^2+^ and Pb^2+^ were both 1 mmol/L, the peak currents were 2.147 nA and 0.721 nA, respectively. When the concentration of Mg^2+^ and Pb^2+^ were both 0.5 mmol/L, the peak currents were 1.398 nA and 0.467 nA, respectively. When the signal-to-noise ratio was 3, the detection limit of Mg^2+^ was 52 μmol/L (the standard deviation of 5 times detection was 2.43%) and the detection limit of Pb^2+^ was 84 μmol/L (the standard deviation of 5 times detection was 3.51%).

## 5. Conclusions 

In this paper, an integrated Ag/AgCl reference electrode structure based on a flow-through amperometric sensor was proposed. The effect of structural sensor parameters (working electrode area, the detection channel height and the sample flow velocity) on the detection signal was investigated by numerically simulation. The simulation results showed that the sample concentration of the electrode surface reduced by 7.0%, when its area was increased by a factor of 1.5 through increasing the micro-channel width. Increasing the area by the same amount through widening the detection cell caused the sample concentration of the electrode surface to reduce by 12.2%. The sample concentration of the electrode surface increased by 1.6%, when the channel height was reduced by half; the sample concentration was maximum when the sample flow rate was 8 × 10^−4^ m/s and the potential driving voltage was 114 V/cm. According to the simulation results, a microfluidic chip using a flow through amperometric sensor as the core was prepared. The chip detection limits of Mg^2+^ and Pb^2+^ were 52 μmol/L and 84 μmol/L, respectively.

## Figures and Tables

**Table 1:** Initial parameters of model.

Name	Value (Unit)	Name	Value (Unit)
Drive potential	10 (V)	Density	1000 (kg/m3)
Dielectric constant	7.08 × 10^−10^ (F/m)	Channel length	3.5 × 10^−4^ (m)
Wall potential	−0.01 (V)	Channel cross section	2 × 10^−^^5^ (m) by 2 × 10^−5^ (m)
Diffusion coefficient	1 × 10^−11^ (m^2^/s)	Sample concentration	1 (mol/L)
